# Blood flow restriction as a post-exercise recovery strategy: A systematic review of the current status of the literature

**DOI:** 10.5114/biolsport.2024.133664

**Published:** 2024-02-07

**Authors:** José M. Oliva-Lozano, Stephen D. Patterson, George Chiampas, Ellie Maybury, Rick Cost

**Affiliations:** 1 United States Soccer Federation. Chicago, IL, United States; 2 Centre for Applied Performance Sciences, St Mary’s University, Twickenham, London, UK

**Keywords:** Ischemia-reperfusion, Ischemic conditioning, Occlusion, Performance, Elite

## Abstract

The aim of this study was to systematically review the current literature on blood flow restriction (BFR) as a post-exercise recovery strategy. Experimental studies investigating the effect of BFR on recovery after exercise were included. Only studies meeting the following inclusion criteria were selected: (a) studies investigating about BFR as a post-exercise recovery strategy in athletes and healthy individuals; (b) the full text being available in English; (c) experimental research study design. Studies that exclusively analyzed BFR as a recovery strategy during the exercise (e.g., recovery strategy between bouts of exercise) were excluded. A literature review was conducted on the PubMed, Cochrane, and Web of Science electronic databases up until May 7^th^, 2023. The main findings were that (i) 9 studies investigated passive BFR as a post-exercise recovery strategy, which shows a significant lack of research in both team and individual sports (especially in female populations), and only 2 studies used active BFR protocols; (ii) although a high quality range of studies was observed, there were methodological limitations such as BFR interventions that were usually conducted after fatiguing protocols or fitness tests, which may not represent the real exercise (e.g., a sprint session of 6 sets of 50 m may induce muscle damage but it does not represent the demands of a team sport like rugby or soccer); (iii) there is a lack of consistency in BFR protocols (e.g., number of cycles or duration of the occlusion-reperfusion periods) for recovery; (iv) some studies showed beneficial effects while others found no positive or detrimental effects of BFR as a post-exercise recovery strategy in comparison with the control/SHAM group. In conclusion, only 11 studies investigated BFR as a post-exercise recovery strategy and there is not any significant amount of evidence in team or individual sports (especially in female populations). BFR could be a potential post-exercise recovery strategy, but practitioners should use caution when applying this method of recovery for their athletes and clients. In addition, it would be of interest for high performance-related practitioners to have a better understanding of the benefits of BFR interventions combined with either active or passive forms of exercise as a post-exercise recovery strategy.

## INTRODUCTION

The duration, intensity, and frequency of exercise contribute to the nature and magnitude of the training effect [1]. Scientific research has become a key piece in the process of understanding training load, which allows to optimize performance, prevent undertraining and overtraining scenarios, and reduce the injury risk [1, 2]. Athletes may experience high levels of stress because of training load, competition demands, travel or lifestyle [3]. Sports such as soccer, basketball, or hockey require high-intensity and multidirectional movements over extended periods during the game, which induce a physiological and metabolic stress [4, 5]. For example, professional soccer players may run 10–12 km per match, being 5–10% at high-intensity [6–8]. Specifically, performing high-intensity accelerations or decelerations may have a significant impact on the players’ mechanical load [9] and muscle damage [10]. Also, these actions are positively correlated with the rating of perceived exertion [11] and neuromuscular fatigue [9].

Currently, athletes are often required to play during periods of fixture congestion, which is defined as a minimum of two successive bouts of match-play with recovery periods of less than 96 hours [12]. Contemporary sport has led to an increase in the number of domestic and international competitions and some of them require playing overtime periods as well [12]. For instance, performance deteriorates 1 to 4 days after a soccer game and acute inflammatory responses were observed with (1) a post-match peak of leukocyte count, cortisol, and cytokines; (2) a 24-hour peak of delayed-onset muscle soreness, C-reactive protein, and thiobarbituric acid reactive substances; (3) a 48-hour peak of creatine kinase, lactate dehydrogenase, and protein carbonyl; and (4) 72-hour peak of uric acid [13]. This has serious implications for sport performance practitioners since players might not achieve complete physical performance recovery during congested schedules [14].

In consequence, efficient recovery strategies are necessary and thus, the use of blood flow restriction (BFR) has been suggested as a strategy to accelerate recovery processes [15]. BFR consists of restricting arterial inflow and completely restricting venous outflow due to the application of a tourniquet or inflatable cuff around the most proximal region of the working upper and/or lower limbs [16, 17]. The cuff’s inflation generates a gradual compression of the vasculature underneath the cuff so the blood flow to structures distal the cuff is restricted, but it more severely affects venous outflow from under the cuff that is proposed to also impede venous return [16]. This compression implies a hypoxia condition within the muscle tissue [18, 19]. From a practical perspective, recent studies recommended the use of BFR combined with various forms of exercise (e.g., resistance exercise, aerobic exercise, or passively) even though other factors such as intensity, volume, restriction time, amount of cuff pressure, size, and cuff material) need to be considered [16, 20, 21].

Although a better knowledge of the underlying mechanisms through which BFR may positively impact post-exercise recovery, the rationale behind the use of this technique may be related to both metabolic and vascular pathways [22]. BFR interventions were initially developed in order to decrease the damage caused to internal organs by ischemia and reperfusion [22, 23] but it may be a useful strategy for performance purposes [22]. For example, the primary mechanisms by which BFR is thought to stimulate muscle growth may include metabolic accumulation which leads to a subsequent increase in anabolic growth factors, fast-twitch fiber recruitment, and more protein synthesis through the mammalian target of rapamycin (mTOR) pathway [24]. In addition, nitric oxide synthase-1 (NOS-1), heat shock proteins, and myostatin have also been shown to be affected by an occlusion stimulus [24]. Also, a different study found that the recovery of functional outcomes after applying BFR post-exercise may be due to a lower decrease in creatine kinase and muscle soreness [25].

However, the benefits of BFR as a strategy to accelerate post-exercise recovery processes are still unclear. Although another review was published in 2020, an update is necessary given the large number of sports performance-related practitioners that are currently using BFR for recovery [15]. In addition, one might wonder if the conclusion of such review (i.e., effective intervention to accelerate performance recovery) is strong enough from a practical application perspective based on the characteristics of the studies that were included. Given that research on the effects of BFR as a recovery strategy is scarce, the aim of this study was to systematically review the current literature on BFR as a recovery strategy in order to investigate the effect of BFR protocols, which were performed after exercise in athletes and healthy individuals, on recovery-related parameters.

## MATERIALS AND METHODS

### Study design

A literature review was conducted on the PubMed, Cochrane, and Web of Science electronic databases up until May 7^th^, 2023. This systematic review was reported in accordance with the Preferred Reporting Items for Systematic Reviews and Meta-Analyses (PRISMA) guidelines [26] and considerations for systematic reviews in sport sciences [27]. The following search strategy by title and abstract was designed:

[“Blood flow restriction” OR “BFR” OR “occlusion” OR “ischemic conditioning” OR “ischemia-reperfusion”] AND [“sport*” OR “exercise” OR “athlete” OR “player” OR “resistance train*” OR “strength train*”] AND [“recovery” OR “cool-down” OR “post-conditioning” OR “post-session” OR “post-training” OR “post-workout” OR “post-match” OR “post-game” OR “post-exercise”].

### Study selection

Only studies meeting the following inclusion criteria were selected: (a) studies investigating about BFR as a post-exercise recovery strategy in athletes and healthy individuals; (b) the full text being available in English; (c) experimental research study design. Studies that exclusively analyzed BFR as a recovery strategy during the exercise (e.g., recovery strategy between bouts of exercise) were excluded.

**FIG. 1:**
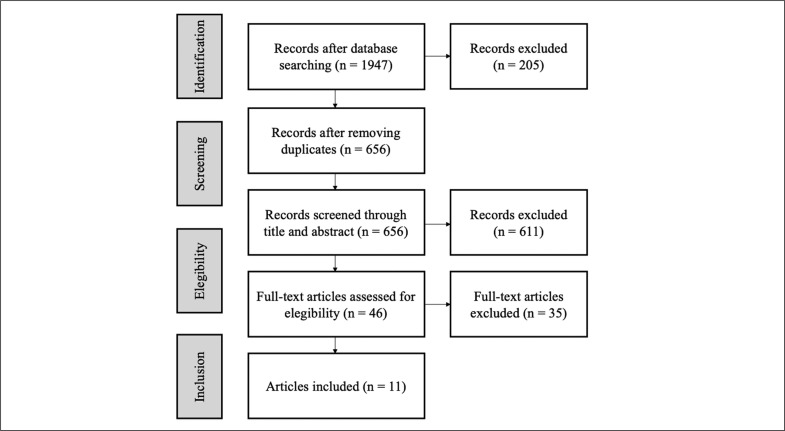
Flowchart of the selection process.

Two independent reviewers selected the studies based on the inclusion and exclusion criteria. All references were stored in the Mendeley reference management system (Elsevier, Amsterdam, The Netherlands). Since duplicates were observed, these were removed. Then, the titles and abstracts were examined. Finally, articles were accessed full text and only studies meeting the inclusion criteria were included in the study. If there was any disagreement between the reviewers, a third collaborator was involved in the decision-making process. A graphical description of the selection process may be observed in Figure 1.

### Data abstraction

The following data were extracted from each study by the reviewers: authors, year of publication, sample size, sample characteristics (e.g., age, gender, or body composition variables), type of exercise protocol or activity, characteristics of the BFR intervention, outcome variables (i.e., recovery-related parameters), and main findings regarding changes in these recovery-related variables after the BFR intervention.

### Methodological quality assessment

The methodological quality of each study was assessed using the PEDro scale [28] if the study had an experimental design with randomly assigned experimental and control groups while the MINORS scale [29] was used for non-randomized trials. The PEDro scale consists of 11 items, which include: eligibility criteria, random allocation, concealed allocation, similarity at baseline, subject blinding, therapist blinding, assessor blinding, > 85% follow-up, intention-totreat analysis, between-group statistical comparison, and point and variability measures [28]. The first item was not included to calculate the total PEDro score as suggested by previous research, so the maximum score was 10 points (low quality: <3; moderate quality: 4–5; high quality: 6–10) [30–32]. When it comes to the MINORS scale, it consists of 12 items (i.e., a clearly stated aim, inclusion of consecutive patients, prospective collection of data, endpoints appropriate to the aim of the study, unbiased assessment of the study endpoint, follow-up period appropriate to the aim of the study, loss to follow up less than 5%, prospective calculation of the study size, adequate control group, contemporary groups, baseline equivalence of groups, and adequate statistical analysis), being the last 4 items specifically for comparative studies [29]. Each item was scored on a scale of 0–2 (0: not reported; 1: reported but inadequate; 2: reported and adequate) for a total score of 24 points (low quality: <8; moderate quality: 9–16; high quality: 17–24) [32]. Any hesitation in the scoring process was resolved by consensus between two independent researchers.

## RESULTS

### Study selection

A total of 1947 studies were identified following the search strategy. Duplicates were removed and a total of 656 titles and abstracts were examined. Of these, 46 were selected for full-text screening. Since 35 studies did not meet the inclusion criteria, 11 studies were selected for the study (Figure 1).

### Characteristics of the selected studies

Table 1 shows the characteristics of the selected studies. These studies have included a total of 206 participants, being 195 male and 11 female participants. The studies collected data from team sports (i.e., soccer and rugby) [33–36], individual sports (i.e., cycling and judo) [37, 38], and other recreational activities in general (i.e., trained/active/healthy individuals) [25, 39–42]. The average age group in these studies ranged between ~17 and ~32 years old.

The exercise protocols were not standardized considering that each study used a different method. However, all BFR interventions were conducted passively with the participant on a supine position and on the proximal portion of the thigh, except for 2 studies that placed the cuffs with active recovery protocols [36, 41]. There were 5 studies that used bilateral BFR [25, 33, 34, 36, 38] while 6 studies used unilateral BFR [35, 37, 39–42]. The cuffs for the experimental groups were usually inflated at 220 mmHg [25, 35, 39, 40] or 50 mmHg above systolic blood pressure [33, 37, 38]. Two studies used 60% of individually calculated pressures [34, 36] and one study used 80% of individual’s resting arterial occlusion pressure [41]. The cuffs for the control/SHAM groups were usually inflated between 15- and 20-mmHg.

Specifically, the BFR protocols consisted of a total of 3 cycles of 5-minute occlusion followed by 5-minute reperfusion or 2 cycles of 3-minute occlusion followed by 3-minute reperfusion [34, 39, 40, 42]. Another study [37] tried two different BFR protocols: 2 cycles of 5-minute occlusion followed by 5-minute reperfusion and 5 cycles of 2-minute occlusion followed by 2-minute reperfusion. Also, two studies included active BFR protocols for recovery (e.g., BFR during the recovery session 24 hours post-match or active protocols including 30 repetitions of biceps curls without external load, followed by 3 sets of 15 repetitions).

**TABLE 1:** Characteristics of included studies.

Reference	Population	Sample size	Sample characteristics	Exercise protocol	BFR intervention	Outcome variables
Arriel et al. (2018) [37]	Cyclists	*n* = 28 (male) Post-exercise ischemic conditioning 2 × 5 group, n = 7 Post-exercise ischemic conditioning 5 × 2 group, n = 7 SHAM 2 × 5 group, n = 7 SHAM 5 × 2 group, n = 7	Post-exercise ischemic conditioning 2 × 5 group: Age = 27.6 ± 4 years old Height: 1.77 ± 0.05 m Weight: 80.3 ± 10.7 kg Post-exercise ischemic conditioning 5 × 2 group: Age = 25.0 ± 4.5 years old Height: 1.76 ± 0.06 m Weight: 76 ± 8.7 kg SHAM 2 × 5 group: Age = 27.8 ± 4.2 years old Height: 1.76 ± 0.02 m Weight: 77.8 ± 7.5 kg SHAM 5 × 2 group: Age = 28.3 ± 2.3 years old Height: 1.75 ± 0.02 m Weight: 74.6 ± 6.2 kg	Maximal incremental cycling test	Post-exercise ischemic conditioning 2 × 5 group (50 mmHg above systolic blood pressure): 2 cycles of 5 min occlusion / 5 min reperfusion. Post-exercise ischemic conditioning 5 × 2 group (50 mmHg above systolic blood pressure): 5 cycles of 2 min occlusion / 2 min reperfusion. SHAM 2 × 5 group (20 mmHg above systolic blood pressure): 2 cycles of 5 min occlusion / 5 min reperfusion. SHAM 5 × 2 group (20 mmHg above systolic blood pressure): 5 cycles of 2 min occlusion / 2 min reperfusion. 5 min after the end of the test, participants adopted a supine position and the occlusion was performed with a blood pressure cuff (77.0 cm length × 21.5 cm width), which was applied unilaterally to the sub-inguinal region of the upper thigh.	Before the incremental test: Perceived recovery scale Muscle soreness Creatine Kinase During the incremental test: Mean heart rate Maximum heart rate Heart rate recovery Peak power output Peak power output Rating of perceived exertion

Beaven et al. (2012) [40]	Recreationally trained individuals	n = 14 (10 males and 4 females) 2 groups of participants following a crossover design: experimental (occlusion) group and control group	Age: 32.0 ± 7.0 years old Body mass: 76.4 ± 12.9 kg	Exercise protocol involving lower-body strength test, power test and repeated sprints	Participants adopted a supine position immediately after the exercise protocol. Unilateral occlusion cuff (BJ Dare Medical Equipment, China), which was placed on the proximal portion of the leg. The cuff had a pneumatic bag along its inner surface which was connected to a pressure gauge that was manually inflated to either 15 mmHg (control group) or 220 mmHg (BFR group) for 3 min. The cuff was alternated with the contralateral leg for an additional period of 3 min. This cycle was repeated twice for a total of 12 min, so both legs had BFR intervention for 6 min per leg.	From CMJ and Squat Jump: Maximum and mean values for eccentric and concentric peak power, peak velocity, and peak acceleration Maximum and mean values for concentric work Jump height Time to peak power and velocity From other tests: CMJ power, squat jump power, leg press maximum strength, leg press average strength, leg press total power, leg press work, leg press velocity, and cumulative sprint times

Castilla-López & Romero-Franco (2023) [36]	Youth soccer players	*n* = 40 (male) 2 groups of participants following a crossover design: experimental (occlusion) group and control group	Age = 17.1 ± 0.8 years old Height: 1.77 ± 0.06 m Weight: 70.2 ± 7.0 kg	Match (inclusion criteria: > 50 minutes played)	Active BFR protocol with players completing a recovery session which consisted of 5-minute running warm-up, 6 high-speed running actions (intensity: 60–70%), and interval drill in the form of rondos with the ball (9 v 2 – 3 × 5 minutes, rest: 90 seconds). This protocol was applied 24 hours after the match. Participants wore the Occlusion Cuff^®^ (Belfast, United Kingdom) with a wrap size (7 × 82 cm in length). The cuffs were placed below the gluteal line in both legs and inflated at the same time. Participants adopted an upright position to set the blood pressure to ~60% of the limb occlusion pressure. The elastic wraps were deflated for 90 seconds between drills.	CMJ height RPE Hooper index

Ceylan et al. (2023) [38]	Elite judo athletes	*n* = 13 (male) 2 groups of participants following a randomized crossover design: experimental group and placebo group	Age: 18.6 ± 0.9 years old Height: 1.74 ± 0.05 m Body mass: 72.4 ± 7.1 kg	Special judo fitness test	Participants adopted a supine position and a pneumatic cuff (77.0 cm length × 21.5 cm width; Riester 5255, Rudolf Riester) was placed around the upper thigh. It was inflated to 50 mmHg above the systolic blood pressure to inhibit arterial flow for 5 min. 3 cycles for each leg with 5 min of reperfusion for each BFR episode. The BFR group achieved a mean pressure of 180 (12) mmHg while the control group was set at 20 mmHg.	Heart rate Lactate Systolic and diastolic blood pressure (mmHg) CMJ height Handgrip strength Muscle soreness

Daab et al. (2021) [33]	Semi-professional soccer players	*n* = 12 (male) 2 groups of participants following a randomized crossover design: experimental group and placebo group	Age = 23.0 ± 1.0 years old Height= 1.79 ± 0.01 m Body mass = 77.9 ± 3.4 kg	Loughborough intermittent shuttle running test	Participants adopted a supine position, and a pneumatic cuff (77.0 cm length × 21.5 cm width) was placed on the proximal portion of the thigh. The protocol consisted of three cycles of 5-minute occlusion and 5-minute reperfusion. Bilaterally vascular occlusion was applied to the BFR group at a pressure of 50 mmHg above the systolic blood pressure and 0 mmHg during the reperfusion. The pressure for the placebo group was set at 20 mmHg.	Squat jump height CMJ height Maximal voluntary contraction of quadriceps (N) 20 m sprint speed Creatine Kinase Lactate dehydrogenase C-reactive protein Muscle soreness

Garcia et al. (2017) [35]	Amateur rugby players	*n* = 8 (male) 2 groups of participants following a randomized crossover design: experimental group and control group	Age = 24.0 ± 4.0 years old Height= 1.79 ± 0.05 m Body mass = 88.0 ± 9.0 kg	8 drills (5 min work per set followed by 30 s passive rest): jumps, skill passing, position scrum with member alternation, slalom agility sprints, rest and hydration, dragon walks, slalom agility sprints, and 20 m sprint	Participants adopted a seated position, and a pneumatic cuff (96 cm length × 13 cm width) was placed on the sub-inguinal portion of the thigh. The protocol consisted of three cycles of 5-minute occlusion, alternated with 2 minutes of reperfusion. The occlusion and reperfusion phases were alternated between thighs. Occlusion was applied to the BFR group at a pressure of 220 mmHg and 0 mmHg during the reperfusion. No cuffs were applied to the control group, which sat passively for 21 minutes.	T-test time CMJ height Continuous CMJ (30 s) height Perceived recovery scale

Lillquist et al. (2023) [42]	Healthy individuals	*n* = 20 (male) 2 groups of participants following a randomized crossover design: experimental group and control group	Age = 21.0 ± 2.8 years old Height= 1.81 ± 0.07 m Body mass = 81.9 ± 13.7 kg	100 box drop jumps (10 sets × 10 repetitions). 10 seconds of recovery was allowed between drops jumps and 1 minute between sets.	BFR was applied with Delfi Portable Tourniquet System (PTS) ii and contoured (12 cm wide), inflation cuff (Delfi Medical Innovations, Inc., Vancouver, BC, Canada). The cuff was placed on the proximal portion of the thigh in each leg and the individualized tourniquet pressure was set as a percentage of limb occlusion pressure, where venous blood flow was completely restricted. session. ~198 mmHg was applied directly to one leg. Post-exercise ischemic conditioning was carried out for 3 sets of 5 minutes on the direct leg (rest between sets: 5 minutes). During the rest period, the opposite leg had 20 mmHg of pressure for 3 sets of 5 minutes per leg (rest between sets: 5 minutes). The cuff was inflated to 20 mmHg for the control group for 3 sets of 5 minutes per leg (rest between sets: 5 minutes).	Muscle soreness Knee flexion and extension peak torque

Northey et al. (2016) [39]	Recreationally trained individuals	*n* = 12 (male) 3 groups of participants following a randomized crossover design: occlusion group, sequential intermittent pneumatic compression, and passive control group	Age: 24.0 ± 6.3 years old Height: 1.80 ± 0.09 m Body weight: 84.8 ± 9.6 kg	100 back squats (10 sets × 10 repetitions) with an initial load of 70% 1RM. If the participant was not able to complete the target number of repetitions without assistance, the load was reduced by 5% of the initial load. There was a 3-minute recovery period between sets.	Occlusion group: participants adopted a supine position with a unilateral occlusion cuff (Flexiport Reusable Blood Pressure Cuff, Welch Allyn, Australia), which was placed on the proximal portion of the leg and inflated to 220 mmHg. 3 min later, the cuff was alternated to the other leg for 3 min, before being repeated on each leg (12 min in total). Sequential intermittent pneumatic compression: participants adopted a supine position with Recovery Boots (RecoveryPump, LLC., USA), which were placed on each leg. The chambers were inflated to a pressure of 80 mmHg with a deflation time of 15 seconds for 45 min. Passive control group: 45 min in a supine position.	Concentric peak isokinetic torque of the quadriceps CMJ height Squat jump height Perceived recovery status Muscle soreness

Page et al. (2017) [25]	Recreationally active individuals	*n* = 16 (male) 2 groups of participants: Experimental group, n = 8 SHAM group, n = 8	Age: 22.6 ± 2.8 years old Height: 1.79 ± 0.06 cm Body mass: 75.5 ± 8.1 kg	100 drop jumps (60-cm height box)	Participants adopted a supine position and bilateral arterial occlusion cuffs were placed on the proximal portion of the thigh (14.5 cm width; Delfi Medical Innovations, Vancouver, Canada). The inflatable cuffs were connected to a pressure gauge and were automatically inflated to 220 mmHg (BFR group) for 5 min followed by 5 min reperfusion. The pressure for the SHAM group was set at 20 mmHg. This procedure was repeated three times (i.e.,15 min of BFR and 15 min of reperfusion).	Creatine kinase Thigh circumference Muscle soreness CMJ height Maximal isometric voluntary contraction

Williams et al. (2018) [34]	Academy rugby union players	*n* = 24 (male) 2 groups of participants following a randomized crossover design: experimental group and control group	Age: 21.8 ± 3.0 years old Height: 1.85 ± 0.09 m Body mass: 96.9 ± 10.1 kg	Sprint session (6 × 50 m)	Participants adopted a supine position and occlusion cuffs (11 cm; Sports RehabTourniquet, Sportsrehab) were applied to the proximal point of the thighs. The cuff was manually inflated to 15 mmHg for the control group while the BFR group’s pressure was set at 60% of individually calculated pressures (171–266 mmHg). The protocol consisted of 2 cycles of 3-min occlusion and 3-min reperfusion.	Salivary testosterone Salivary cortisol Blood lactate Creatine Kinase Perception of muscle soreness Peak power output CMJ height

Wong et al. (2022) [41]	Healthy individuals	n = 19 (12 male and 7 female) Each arm was randomly assigned to BFR group or SHAM. Each participant completed both conditions.	Age: 22.0 ± 3.0 years old Height: 1.71 ± 0.09 m Body mass: 76.9 ± 16.4 kg	4 sets × 90 bilateral biceps curls (Male: 4.08 kg; Women: 2.18 kg). If participants could not keep a 60-beat per minute pace, a lighter load was used. Rest: 30 s between sets	Active BFR protocol with both arms simultaneously performing 30 repetitions of biceps curls, followed by 3 sets of 15 repetitions. Rest: 30 seconds of rest between sets. 5 cm nylon cuffs (SC5 Hokanson, Bellevue, WA) were placed on the most proximal portion of both arms. Each arm was randomly assigned to either the blood flow restriction or the SHAM treatment. The cuff of the BFR group was inflated to 80% individual’s resting arterial occlusion pressure while the other arm served as a sham (0 mmHg). The BFR intervention was performed 5 min and 24 hours after the fatiguing protocol.	Each arm’s maximal isometric torque

**Note:** BFR = Blood flow restriction; CMJ = Countermovement jump; RM = repetition maximum; RPE = rating of perceived exertion

Regarding the outcome variables that could be associated with the recovery status, the studies analyzed parameters related to muscle soreness, perceived recovery status, perceived effort, neuromuscular function (e.g., strength, speed, or power), and physiological markers (e.g., creatine kinase, salivary cortisol, lactate dehydrogenase, or C-reactive protein).

Finally, the methodological quality assessment showed that the PEDro scores for the randomized trials [25, 33–37, 39–42] were ~7.6 (range 6–10), which may be considered as high quality. Also, only one of the studies followed a non-randomized protocol, so the MINORS scale was used and the score was 21 out 24, which represents a high quality score [40]. The criteria with the lowest scores in both scales were those related to random allocation, concealed allocation, and assessor/therapist/participant blinding.

### Main findings related to BFR interventions in team sports

Two studies analyzed the effect of BFR on the recovery process in semiprofessional soccer players [33] and youth players [36], and two BFR interventions were conducted on academy and amateur rugby union players [34, 35]. The youth soccer players did not get beneficial effects from the active BFR protocol for recovery of jumping ability or perceived wellness (i.e., Hooper index). In fact, the BFR group, which wore the cuffs during the recovery session, had increased ratings of perceived exertion right after the session [36]. However, the study with semiprofessional soccer players found that the BFR group got to attenuate the increases in muscle damage markers and muscle soreness in comparison with the placebo group [33]. Also, the BFR group got to attenuate the decrease of squat jump and CMJ height in 24 hours [33]. In addition, BFR could accelerate post-exercise recovery of sprint performance and maximal voluntary contraction of quadriceps [33]. Nevertheless, BFR had no effect on perceived recovery, neuromuscular or physiological markers after the exercise protocol and BFR in the academy or amateur rugby players [34, 35].

### Main findings related to BFR interventions in individual sports

One study analyzed the effect of BFR on the recovery process in cyclists with ~3.2 years of training experience [37] and another BFR intervention was conducted on elite judo athletes [38]. Both studies found beneficial effects of BFR as a recovery strategy compared to the SHAM groups. For example, BFR prevented a decrease in performance 24 hours after the incremental cycling test, which may be due to a late effect of the BFR [37]. Creatine Kinase and muscle soreness were similar between the groups of cyclists (baseline and 24 hours post exercise) [37]. However, perception of recovery scores showed that the post-exercise ischemic conditioning groups felt more tired 24 hours after BFR [37].

In judo athletes, the BFR group had a decrease in heart rate at 30- and 60-minutes during recovery [38]. In addition, CMJ performance in the BFR group was better at 60 minutes compared to the control group [38]. Moreover, the BFR group reported lower muscle soreness than the control group [38].

### Main findings related to BFR interventions in recreationally active individuals

The use of BFR as a recovery strategy has been investigated in other recreational activities (i.e., trained/active/healthy individuals) [25, 39–42].

One of the studies showed that BFR interventions may accelerate the recovery process since a return to pre strength levels 24 hours earlier than the SHAM group was observed [25]. This recovery of functional outcomes may be due to a decrease in the inflammatory response observed after strenuous eccentric exercise because of reduced creatine kinase and muscle soreness [25]. In this line, a different study found that BFR interventions had a significant but marginal effect on mitigating perceptual quadriceps muscle soreness ratings 24 hours after the drop jump fatiguing protocol; however, there was no effect on muscle strength [42].

However, another study concluded that the occlusion and sequential intermittent pneumatic compression groups did not further improve recovery of muscular performance after a fatiguing exercise protocol compared to a passive control group [39]. Perceived recovery and muscle soreness were not significantly different in comparison with the control group [39]. Nonetheless, participants reported that they preferred the “novel recovery interventions” [39].

A third study on recreationally active individuals observed that BFR intervention had positive and negative effects on specific variables related to the neuromuscular function [40]. On the one hand, BFR had a positive effect on the average squat jump height right after the intervention compared to the control group [40]. Specifically, most positive effects of BFR were found 24 hours after the intervention [40]. For example, there was a likely positive effect on the rate of recovery of maximal power production (W) in the squat jump in comparison with the control group. Regarding the squat jump, there were also large positive effects of the BFR on recovery of eccentric peak power and eccentric peak acceleration. Also, the BFR had a beneficial effect in comparison with the control condition on the mean concentric and eccentric peak velocity during CMJ. Moreover, the total power generated in the leg press test showed better results for the BFR group 24 hours post intervention. In addition, the BFR group recovered at a better rate in comparison with the control group when the cumulative 10 m and 40 m sprint times were analyzed 24 hours after the intervention.

On the other hand, there were likely detrimental effects on mean eccentric peak velocity and acceleration in the CMJ and mean eccentric peak power in the squat jump immediately after the BFR intervention [40]. Other immediate effects were generally unclear or trivial [40]. Likely detrimental effects of the BFR were found in the change in rate of recovery on the mean concentric work generated in the squat jump 24 hours after the intervention [40]. Other delayed effects were generally unclear [40].

Finally, another study used an active BFR protocol [41] in which each arm was randomly assigned to either the blood flow restriction or the SHAM treatment (0 mmHg). The BFR intervention was performed 5 min and 24 hours after the fatiguing protocol, but the results indicated that this type of intervention did not improve recovery [41].

## DISCUSSION

The purpose of this study was to review the current literature on the use of BFR as a post-exercise recovery strategy. The main findings were that (i) only 9 studies investigated BFR as a post-exercise passive recovery strategy, which shows a significant lack of research in both team and individual sports (especially in female populations), and only 2 studies used active BFR protocols; (ii) although a high quality range of studies was observed, there were methodological limitations such as BFR interventions that were usually conducted after fatiguing protocols or fitness tests (e.g., incremental cycling test or sprint tests), which may not represent the real exercise (e.g., a sprint session of 6 sets of 50 m may induce muscle damage but it does not represent the demands of a team sport like rugby or soccer); (iii) there is a lack of consistency in BFR protocols (e.g., number of cycles or duration of the occlusion-reperfusion periods) for recovery; (iv) some studies showed beneficial effects while others found no positive or detrimental effects of passive BFR as a post-exercise recovery strategy in comparison with the control/SHAM groups.

This review observed that there is lack of research in team and individual sports. Only 2 studies were conducted in team sports like soccer or rugby, and only 1 study for individual sports like judo or cycling, so it is difficult to draw solid conclusions about the benefits of BFR as a post-exercise recovery strategy. In addition, the methodology of these investigations has several limitations such as the sample characteristics (e.g., lack of research on multiple populations like professional and amateur athletes, or female samples), assessor/therapist/participant blinding (perhaps due to the nature of the intervention), or the exercise protocols that have been used to induce fatigue. For instance, one of the studies in soccer players used the Loughborough intermittent shuttle running test, which is a field test that simulates the activity pattern of soccer [43] However, actions such as running backwards, jumping, or time in possession of ball that are not included and some of these activities along with tackles and sudden changes of direction require eccentric contractions, which may increase the neuromuscular demands imposed by match in comparison with Loughborough intermittent shuttle test [44]. In addition, this kind of data collections is not contextualized to elite level scenarios, in which soccer players may experience successive matches (e.g., less than 96 hours between games) in congested calendars [12]. In consequence, research on players which use BFR as a recovery strategy and is conducted in the realm of current soccer is necessary. Furthermore, there may be different forms of recovery (e.g., active and passive, or post-training session and post-match) and different variables that represent the level of recovery. Based on the studies included in this review, recovery may be understood from multiple perspectives (e.g., changes in muscle damage parameters, perceived recovery or muscle soreness, neuromuscular performance in readiness tests such as countermovement jump or isometric voluntary contractions, etc.) and different recovery protocols have been included, which may explain why some studies got positive effects and others did not.

Although BFR interventions were usually conducted with the participant on a supine position and on the proximal portion of the thigh, this literature review also found that there is a lack of consistency in BFR protocols for recovery. For example, the cuffs for the experimental groups were usually inflated at 220 mmHg [25, 39, 40] or 50 mmHg above systolic blood pressure [33, 37, 38]. In this regard, a previous review suggested that absolute tourniquet pressures from 200 to 250 mmHg have been often used regardless of body size [45]. One of the studies explained that they use a novel technology for BFR which determined the individual personal tourniquet pressure as a percentage of limb occlusion pressure, where venous blood flow was completely restricted [42]. There were 2 studies using 60% of individually calculated pressures [34, 36] and another study using 80% of individual’s resting arterial occlusion pressure [41]. This shows that to date, there has been a lack of standardization of pressure for BFR and recovery. A previous study suggested that it might be recommended to establish pressures based on measurements of arterial occlusion pressure, with pressures ranging from 40 to 80% of arterial occlusion pressure [16]. The reason is that the arterial occlusion pressure is related to a wide range of individual characteristics (e.g., size of the limb, individual’s blood pressure, or tourniquet shape, width, and length) [16, 46, 47]. However, this range of 40 to 80% of arterial occlusion pressure would make more sense for BFR with exercise and the total occlusion pressure (i.e., 100%) may be more closely related to BFR for recovery. In addition, a previous systematic review observed that some of the studies analyzing the effect of BFR on recovery did not clearly describe the characteristics of the participants and expressed concern about the statistical analysis [15]. Consequently, these limitations have a significant effect on understanding the benefits of BFR as a post-exercise recovery strategy.

Another main finding of this literature review was that some studies observed beneficial effects of BFR on the recovery process while others found no positive or detrimental effects of BFR as a post-exercise recovery strategy in comparison with the control/SHAM group. A total of 5 studies highlighted some beneficial effects of BFR on recovery [25, 33, 37, 38, 40], but unclear or detrimental effects were observed in 6 studies [34–36, 39, 41, 42]. In this regard, a previous investigation concluded that BFR interventions could lead to overall faster performance recovery, lower creatine kinase increase, and lower muscle soreness over 24 hours [15]. In addition, it has been suggested that the effectiveness of this intervention may be more specific to low/moderate fitness level individuals [15] and the mechanisms that explain the benefits of BFR for improving recovery and performance are unclear, but likely involve changes in both vascular and metabolic pathways [48]. This inconsistency among studies might be also due to the fact that recovery may be understood from multiple perspectives (e.g., changes in physiological parameters such as creatine kinase or modifications of perceived recovery and neuromuscular performance-related variables).

This review has several limitations to acknowledge. For example, the lack of studies, heterogenous methodologies, and the discrepancy between the results on the benefits of BFR as a post-exercise recovery strategy make it difficult to draw solid conclusions. For example, specification of cuff width and material should be included by each study as these variables may impact the effectiveness of BFR interventions [16]. Also, studies on BFR for recovery were mainly focused on passive strategies, so future research is not only necessary in this direction but also considering active strategies [16]. In this regard, the authors from a previous study recommended using BFR combined with various forms of exercise (e.g., resistance or aerobic) [16].

## CONCLUSIONS

BFR could be a potential post-exercise recovery strategy, but practitioners should use caution when applying this a method of recovery for their athletes and clients. Only 11 studies investigated BFR as a post-exercise recovery strategy and there is not any significant amount of evidence in team or individual sports (especially in female populations). Some studies showed beneficial effects while others found unclear or detrimental effects of passive BFR as a post-exercise recovery strategy in comparison with the control/SHAM groups. Also, a lack of consistency in BFR protocols (e.g., number of cycles or duration of the occlusion-reperfusion periods) for recovery was observed.

In addition, this study has implications for practice and future research. Although limited data are available, the BFR protocols that have shown potential positive effects on recovery markers may be beneficial for specific populations [25, 33, 37, 38, 40]. These protocols may be found in Table 1. For example, soccer players may adopt a supine position, use bilateral BFR (i.e., cuff placed on the proximal portion of the thigh: 77.0 cm length × 21.5 cm width) for 3 cycles of 5-minute occlusion and 5-minute reperfusion at a pressure of 50 mmHg above the systolic blood pressure and 0 mmHg during the reperfusion. Moreover, athletes from cyclic sports such as cycling may use BFR (i.e., cuff applied unilaterally to the sub-inguinal region of the upper thigh: 77.0 cm length × 21.5 cm width) in a supine position for 2 cycles of 5 min occlusion and 5 min reperfusion or 2 min occlusion and 2 min reperfusion at a pressure of 50 mmHg above systolic blood pressure.

Future studies need to analyze the benefits of BFR for recovery including sport-specific protocols (e.g., after participation in a full game or competition), and considering contextual variables (e.g., congested fixture periods) and actual protocols (e.g., pressures individualized to arterial occlusion pressure). In addition, it would be of interest for high performance-related practitioners to have a better understanding of the benefits of BFR interventions combined with either active or passive forms of exercise as a post-exercise recovery strategy. Another thing to consider is that passive BFR and BFR with exercise influences pain so could be used to help those recover if players have pain and niggles post exercise [49–51].

The use of BFR as a recovery strategy may be considered by practitioners, but the level of applicability to each specific context needs to be analyzed as well. For example, the timing of the intervention in high performance sports is very important, especially when there is travel involved after the competition. Also, further research is neeed to understand if it is likely that a single bout of restriction can improve recovery for the next days based on the initial change in blood flow. Furthermore, knowing the estimated duration of the effect of a BFR intervention would help understand the evolution of the recovery process. Nonetheless, it is necessary to gain a better understanding of the mechanisms of BFR for recovery. For instance, if the potential beneficial effects of BFR on exercise performance and recovery can be linked to post occlusion increases in blood flow associated with elevated adenosine levels and activation of ATP-sensitive potassium channels, which may lead to increased blood flow and improved skeletal muscle contractile function [39, 40]. In this regard, future research will be necessary to understand the effects of BFR on recovery markers.

## Conflict of Interest Disclosure

The authors declare no conflicts of interest. This manuscript is original and not previously published, nor is it being considered elsewhere until a decision is made as to its acceptability by the journal.
